# Quaternary Ammonium Chitosans: The Importance of the Positive Fixed Charge of the Drug Delivery Systems

**DOI:** 10.3390/ijms21186617

**Published:** 2020-09-10

**Authors:** Angela Fabiano, Denise Beconcini, Chiara Migone, Anna Maria Piras, Ylenia Zambito

**Affiliations:** 1 Department of Pharmacy, University of Pisa, Via Bonanno 33, 56126 Pisa, Italy; angela.fabiano@unipi.it (A.F.); denisebeconcini@gmail.com (D.B.); chiara.migone@for.unipi.it (C.M.); anna.piras@unipi.it (A.M.P.); 2 Interdepartmental Research Centre “Nutraceuticals and Food for Health”, University of Pisa, 56100 Pisa, Italy

**Keywords:** trimethyl chitosan, *N*-2-hydroxypropyl trimethyl ammonium chitosan, dimethylethyl chitosan, diethylmethyl chitosan, quaternary carboxymethyl chitosan derivatives, in vitro studies, ex vivo studies

## Abstract

As a natural polysaccharide, chitosan has good biocompatibility, biodegradability and biosecurity. The hydroxyl and amino groups present in its structure make it an extremely versatile and chemically modifiable material. In recent years, various synthetic strategies have been used to modify chitosan, mainly to solve the problem of its insolubility in neutral physiological fluids. Thus, derivatives with negative or positive fixed charge were synthesized and used to prepare innovative drug delivery systems. Positively charged conjugates showed improved properties compared to unmodified chitosan. In this review the main quaternary ammonium derivatives of chitosan will be considered, their preparation and their applications will be described to evaluate the impact of the positive fixed charge on the improvement of the properties of the drug delivery systems based on these polymers. Furthermore, the performances of the proposed systems resulting from in vitro and ex vivo experiments will be taken into consideration, with particular attention to cytotoxicity of systems, and their ability to promote drug absorption.

## 1. Introduction

The current research in the field of controlled drug delivery systems has been focused on the use of polymeric materials. These, due to their unique properties, they can be easily modified and hence utilized in the pharmaceutical and food or cosmetic industry [1].

Since synthetic polymeric materials often cause side effects, natural polymeric materials, extracted from starch, inulin, cellulose, chitin or alginates, are preferred for use as excipients (e.g., binders, viscosity enhancers etc.) for the preparation of controlled drug delivery systems [2]. Among the natural polymers, chitosan is one of the most used and its safe application in the pharmaceutical and food industry has been approved by the U.S. FDA. 

Chitosan is a cationic polysaccharide of d-glucosamine with some *N*-acetyl-d-glucosamine residues linked by β (1-4) bonds. It has excellent biological properties such as biocompatibility, biodegradability and mucoadhesivity moreover it has antimicrobial, antiviral and immunoadjuvant activities. Chitosan occurs rarely in nature and it is obtained by incomplete deacetylation of chitin that is a homo-polymer of β (1-4) linked units of *N*-acetyl-d-glucosamine, present in the shell of crustaceans and molluscs, cuticle of insects and cellular walls of fungi. Chitosan is able to enhance drug penetration not only through cell monolayer epithelia such as intestinal [3,4] and nasal [5], but also through stratified epithelia such as buccal [6,7], vaginal [7] and corneal tissue [8,9,10,11].

For all these characteristics, chitosan has been used for the preparation of conventional pharmaceutical systems (e.g., solutions, suspensions, emulsions, etc.) and for the development of innovative drug delivery systems such as colloidal systems and hydrogels.

**Figure 1:**
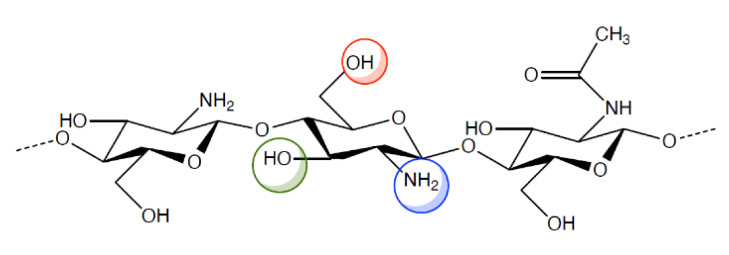
Chemical structure of chitosan and its functional groups.

However, the pharmacologic and therapeutic application of chitosan is limited by its insolubility in water and in most organic solvents. For this reason, various chemical modifications of chitosan molecular structure have been made in order to increase polymer solubility and, hence, its applications. Indeed, chitosan (Figure 1) has active groups on its backbone, such as -OH, -NH_2_, that can be modified to generate different derivatives. The resulting chitosan derivatives have the same properties as the parent polymer, only, they have enhanced biocompatibility and non-toxicity [12,13,14,15,16].

A great deal of different chitosan derivatives, including acylated, alkylated, carboxylated and ammonium quaternary chitosan conjugates have been described. It is known that even slight physical-chemical differences in the polymer backbone are reflected in significant biological differences concerning, for example, cellular absorption, the biological processes that regulate this absorption and the interaction with the mucous lining of epithelia. Indeed, numerous studies show that drug release systems based on chitosan and its derivatives promote the absorption and biodistribution of drugs in a manner strictly dependent on the properties of the polymer. These properties could then be modulated to make cellular absorption selective, thus targeting the transported drug to the site of action, which would result in stronger pharmacological activity with less systemic exposure. In this review we will focus on the quaternary ammonium derivatives of chitosan in order to evaluate the impact that the positive fixed charge has on the biopharmaceutical characteristics of the relevant drug delivery system.

## 2. Quaternary Ammonium Chitosan Derivatives: Principal Characteristics and Applications

A large number of chitosan derivatives have been prepared via alkylation, quaternization, carboxylation, phosphorylation and sulfation, in order to increase chitosan solubility and extend its application in drug delivery systems. Among chitosan derivatives, quaternary ammonium salts have been the most used in tissue engineering, drug and gene delivery and wound care [17].

Chitosan derivatives are generally obtained by modifications that do not involve the basic chemical structure of chitosan, but yet they lead to compounds with changed, improved properties. Quaternary ammonium chitosan derivatives are readily water soluble irrespective of pH, therefore they enhance the release and the permeation of drugs across biological barriers in neutral/alkaline environments [18]. In particular, these derivatives have a permanent positive charge, an enhanced mucoadhesivity and a high drug loading capacity, in addition to biocompatibility, low toxicity and biodegradability. For these reasons, they are optimal candidates for the development of conventional and innovative systems delivering drugs through different routes of administration, as shown in Table 1 and as will be discussed in the following sections [1]. 

In virtue of their antibacterial activity, chitosan derivatives can be also used as anti-inflammatory drugs or as fibre fillers for wound dressings [19,20,21,22,23].

### 2.1. N-2-Hydroxypropyl Trimethyl Ammonium Chloride Chitosan Derivative (HACC)

HACC is a quaternary ammonium salt widely used in recent years as an excipient for controlled drug or gene release. HACC can be obtained through various synthetic routes. Peng et al. [68], obtained HACC by reaction of chitosan with glycidyl trimethylammonium chloride (GTMAC). GTMAC is a small quaternary ammonium molecule with an epoxy group that reacts easily with the amino groups on the chitosan backbone.

Another synthetic route, reported by Jin et al. [69], involves the reaction of chitosan with 2,3-epoxypropyl trimethyl ammonium chloride (EPTAC). This synthesis was subsequently improved with the introduction of the concept “green chemistry”, through the use of an ionic liquid, 1-allyl-3-methylimidazole chloride, as the reaction solvent [70].

HACC represent a promising biomaterial for the preparation of nanoparticles (NPs), wound dressings and hydrogels, because it is non-cytotoxic and has better antimicrobial and antibacterial properties than unmodified chitosan [71,72,73,74,75].

In particular, Ao et al. [24], exploited the antibacterial activity of HACC and used it to prepare wound dressing systems. The authors demonstrated that wound dressings with good antibacterial properties and biocompatibility could be obtained by optimizing the concentration and the degree of substitution (DS) of HACC in bacterial cellulose culture medium.

Fan et al. [25], prepared HACC hydrogel using gamma radiation and demonstrated its potential application as a scaffold in wound healing. Indeed, the in vitro studies revealed a strong inhibitory effect of HACC hydrogel against *Staphylococcus aureus* and *Escherichia coli*, thus proving its antibacterial activity.

The ability of HACC to provide a thermosensitive and reversible sol gel transition is also very interesting. It was studied by Wang et al. [26], who prepared liposomes-containing thermosensitive hydrogels based on HACC and glycerophosphate, medicated with doxorubicin. The release of doxorubicin from liposomes-containing hydrogels in nine days was about 22% of the drug load. In vivo tests on rats showed that tumour growth in the doxorubicin group was significantly inhibited. However, serious side effects were observed. The weight of the mice in the doxorubicin group decreased significantly. The side effects were reduced by encapsulating doxorubicin in liposomes, but anticancer activity was also slightly reduced. Introduction of medicated liposomes into the HTCC-based gel significantly improved the antitumor doxorubicin activity.

This polymer was found to be suitable for preparing nanosystems such as polymeric NPs. In particular, Jin et al. [27], prepared HACC-based NPs by ionotropic crosslinking with carboxymethyl chitosan (CMC) as a carrier of Newcastle disease virus (NDV) [69]. HACC has shown good antimicrobial and antifungal activity in itself, coupled with NDV activity. Moreover, the application of HACC derivatives as nanocarriers able to enhance the immune response and the efficacy of vaccines has been demonstrated. 

Lu et al. [28], successfully prepared HACC NPs loaded with paclitaxel (PTX) for the oral administration of this anticancer drug. These authors used in vitro, ex vivo and in vivo experiments to compare the behaviour of two NPs types, one based on HACC the other based on unmodified chitosan. The results showed a better intestinal permeability and cellular absorption as well as a more effective inhibition of tumour growth and induction of apoptosis in cancer cells with the former NP type. These results, which were ascribed to the presence of fixed positive charges on these NPs, highlight the importance of positive fixed charges on the absorption and internalization of polymer nanosystems.

Furthermore, HACC NPs are currently considered useful carriers for the oral delivery of hydrophilic molecules, such as proteins and peptides. Indeed, it was demonstrated that NPs based on HACC and coated with thiolated hyaluronic acid were able to promote the oral delivery of insulin, thanks to the high mucus-penetration ability of the coating [29].

Recently, Li et al. [30], proposed the synthesis of new fatty acid modified HACC, prepared by the conjugation of lauric acid or oleic acid to the quaternized polymer. These two derivatives were used to prepare NPs to deliver insulin to liver. The data showed that the NPs obtained by the more aliphatic derivative, that is oleic acid quaternized chitosan, were more internalized by liver cells than all the other NPs tested and this resulted in a more relative pharmacological availability.

### 2.2. N-Trimethyl Chitosan (TMC)

The TMC polymer has widely been used for the delivery of drugs and genes [76] through different routes, e.g., peroral [77], ocular [78], nasal [79], buccal [80], pulmonary [81] and rectal [82]. Moreover, TMC has been widely used in medicine, food industry and other fields [83].

The most common method for its preparation involves methylation by methyl iodide. However, the resulting polymer, TMC iodide, is unsuitable for pharmaceutical or cosmetic purposes, because it is very toxic for ingestion or inhalation. For this reason, this salt has to be converted into TMC chloride in the final purification step, by means of the dialysis technique. In addition, a number of alternative reaction types have been developed well reported in a previous review [84]. Wu et al. [85], synthesized TMC by applying the concept of “green chemistry”, i.e., by reacting chitosan with dimethyl carbonate in the presence of the ionic liquid, 1-butyl-3-methylimidazolium chloride as catalyst. Very recently, Rathinam et al. attempted a selective methylation of chitosan from tert-butyldimethylsilyl-chitosan in a multi-step process, which involved protection with Boc. A derivative was obtained in which part of the primary amino groups have been trimethylated and the remaining amino groups have not been modified [86].

TMC like chitosan, has muco-adhesive properties that depend on the charge density and have been attributed to an interaction between the cationic groups of TMC and the anionic sialic and sulfonic acid residues of mucin. TMC promotes the transport of hydrophilic and peptide molecules through the paracellular route, as it is able to open the tight junctions between epithelial cells [84,86,87,88,89].

TMC salts, including *N*,*N*,*N*-trimethylchitosan citrate, *N*,*N*,*N*-trimethylchitosan ascorbate, *N*,*N*,*N*-trimethylchitosan acetylsalicylate, *N*,*N*,*N*-trimethylchitosan gallate etc., were synthesized to improve the chitosan antioxidant power. All these derivatives have both antioxidant and radical scavenging activity, especially ascorbate and gallate salts [90].

Thanks to its amphiphilic nature, TMC can be assembled into vesicles that can be used in nanomedicine for the preparation of several innovative drug delivery systems for pharmaceutical, biomedical and biotechnological applications [91]. Numerous TMC-based colloidal systems have been reported in the literature such as, e.g., TMC-based polyelectrolyte nanocomplex, TMC-based nanoparticles, TMC-based liposomes, etc., as efficient drug delivery systems for the treatment of various forms of cancer, hypertension, rheumatoid arthritis as well as peptide, gene and vaccine delivery.

In particular, Sayin et al. [31], synthesized TMC-based NPs loaded with tetanus toxoid. When compared with NPs based on unmodified chitosan or on carboxymethyl chitosan (MCC), this NPs type demonstrated, either in vitro or in vivo, their safety, ability to be uptaken by cells and induce immune responses. Very interestingly the chitosan and TMC NPs, which have positively charged surfaces, induced higher serum IgG titrers than those prepared with MCC, which are charged negatively.

Sandri et al. [32], evaluated TMC-based NPs as carriers for the oral administration of insulin and demonstrated that, thanks to their high mucoadhesivity, they were internalized by duodenum and jejunum cells much more than chitosan-based NPs.

Similar results were obtained by other authors [33], who showed that TMC-PLGA NPs, thanks to their positively charged surfaces, can improve penetration through mucus, uptake and permeation of insulin through the intestinal epithelium much more than the uncoated PLGA NPs. Furthermore, it was demonstrated that the mucoadhesive, targeted PLGA NPs, surface-modified by lactoferrin-conjugated TMC, promote intranasal drug delivery to the brain and can be used for Alzheimer’s disease treatment [34]. On their part Rassu et al. [35], used TMC alone for the preparation of particulate systems for nose-to-brain drug delivery and treatment of central nervous system diseases.

TMC-based polyelectrolyte complexes for DNA delivery were prepared by electrostatic complexation between pDNA and TMC. Zheng et al. [37], demonstrated that the cellular uptake of folate-TMC/pDNA nanocomplex was higher than that of TMC/pDNA nanocomplex thanks to the folate receptor mediated endocytosis.

TMC has also been used for the preparation of several liposomal systems [38]. TMC-coated liposomes for the oral delivery of drug and natural compounds have been developed. Their efficacy in promoting absorption and controlling release of molecules, e.g., harmine, calcitonin, curcumin, was demonstrated by both in vitro and in vivo studies [39,40,41,42].

TMC finds another interesting application in wound-healing, along with such wound dressing materials as films, fibres, hydrogels, etc. Among the polymers used, such as collagen, alginate and chitosan, chitosan represents the safest material for wound dressing. However, chitosan fibres have poor liquid absorbing properties and antibacterial activity. To overcome these limits, Zhou et al. [66], prepared TMC fibres with various quaternization degrees, which exhibited higher absorption promotion ability and antibacterial properties compared to those based on chitosan, as shown by in vitro and in vivo studies. Indeed, Rúnarsson et al. synthesized a series of methylated derivatives of chitosan with different methylation degrees and found them active against *S. aureus*, even at pH 7.2 at which the unmodified chitosan was inactive, thus demonstrating the importance of the fixed positive charge for the antibacterial effect of polymers [43].

The enhanced antibacterial and antimicrobial activity of TMC was also used to prepare hydrogels and blends. For example, Mohamed et al. [92], synthesized hydrogels based on TMC, cross-linked with poly(vinyl alcohol) (PVA) to increase the antimicrobial and antibacterial activity of the latter. Noticeably Boles et al. [93], prepared injectable local delivery systems based on a combination between TMC and poly(ethylene glycol) diacrylate chitosan, medicated with vancomycin and amikacin. The resulting blend was not-cytotoxic and improved the antimicrobial activity of drugs.

Other administration routes involving TMC-based delivery systems have been explored. For example, TMC and its derivatives have been used in nasal delivery systems, especially as a base material for forming micro- and nanoparticles, for therapeutic applications [36].

The preparation of TMC derivatives containing cysteine (TMC-Cys) was proposed as a strategy to increase TMC mucoadhesivity [94]. TMC-Cys, which was synthesized through the formation of amide bonds between the non-substituted primary amino groups of TMC and the carboxylic groups of cysteine, combined the mucoadhesive characteristics of thiomers with the advantages of the fixed positive charges of TMC. In fact, Zhao et al. [45], demonstrated that the positive groups present on TMC and the mucoadhesive characteristics of thiol groups favour the interaction of TMC-Cys with the cell membrane, thus enhancing cellular uptake.

Thanks to their mucoadhesion and permeation-enhancing properties, the TMC-Cys derivatives have been used for the preparation of such safe and efficient oral delivery systems as NPs. For example, Yin et al. [94], synthesized TMC-Cys NPs loaded with insulin. Oral and ileal administration of TMC-Cys NPs led to more significant hypoglycaemic effects than those of the insulin solution. In addition, with this NPs type a stronger and longer-lasting hypoglycaemic effect was observed compared to TMC NPs, which is in agreement with the mucoadhesion and permeation improvement results.

TMC-Cys was also able to form nanocomplexes for gene delivery [45,46]. The authors demonstrated that the positive charge of TMC-Cys would interact with the negative charge of pDNA, thus stabilizing the complexes and hence, protecting the pDNA from degradation that occurs during the extracellular transit. Nanocomplexes based on TMC-Cys and condensed with DNA were prepared also by Rahmani et al. [47]. The thiol groups present on the TMC-Cys conjugate enhance the transfection efficiency, thus making efficient gene delivery systems.

Also, a gene silencing activity of siRNA polyplexes based on TMC-Cys was demonstrated, that was much higher than that of complexes based on non-thiolated TMC [48]. These authors showed that the introduction of thiol groups in the TMC chain enhances the extracellular stability of the TMC-Cys complexes, due to formation of reducing disulfide bonds, and promotes the intracellular release of siRNA. Hence, TMC-Cys could represent a promising vector for gene delivery thanks to the fixed positive charges and the thiol groups which co-operate to improve the properties of unmodified chitosan.

### 2.3. Quaternary Carboxymethyl Chitosan Derivative (QCMC)

QCMC are water-soluble chitosan derivatives containing anionic and cationic groups. They possess antimicrobic activity [95,96,97], flocculating properties [98] and antioxidant activity [99]. By virtue of their properties, QCMC are used in various fields, such as tissue engineering, food and cosmetic industry, as well as antioxidant, antimicrobial and drug delivery systems.

Sun et al. [100], synthesized QCMC by reacting CMC with 2,3-epoxypropyl trimethylammonium. The resulting QCMC derivatives possess a strong antimicrobial activity due to a synergistic effect of carboxylic and quaternary ammonium groups present on the chitosan backbone. Li et al. [101], prepared QCMC with antioxidant activity starting from a reaction between chitosan and chloroacetic acid using 2,3-epoxypropyltrimethyl ammonium chloride as a modifying agent under microwave irradiation. These authors demonstrated that quaternary ammonium and carboxylic groups have a different impact on the polymer antioxidant activity, in fact the former, while on the one hand enhancing the OH scavenging activity, on the other hand exert a negative effect on the polymer metal chelating ability. Both these properties depend on the degree of substitution (DS) of quaternary ammonium and carboxyl groups on polymer chain. Indeed, the authors found that a high DS of quaternary ammonium groups lowers the reducing power, whereas a moderate DS of carboxyl groups enhances it.

Concerning the use of QCMC as antimicrobial and antibacterial agents Yin et al. [49], prepared blend films based on QCMC and PVA loaded with Cu^2+^, as potential biomaterials for biomedical application, with good antibacterial activity, in fact, they inactivated 98.3% of *S. aureus* and 99.9% of *E. coli.* For the same application, Huang et al. [50], synthesized silver nanoparticles in an aqueous solution of QCMC, used as a chemical reducing and stabilizing agent. These nanoparticles had a better antibacterial property and a lower toxicity than a solution of QCMC. Liang et al. [51], prepared liposomes from QCMC derivatives containing cholesterol and medicated with vincristine. These liposomes had a structure similar to conventional liposomes prepared from phosphatidylcholine/ cholesterol, but they had better thermal stability, water solubility and drug loading efficiency. Moreover, they exhibited a steady drug release within 2 weeks at pH 7.4.

### 2.4. Dimethyl Ethyl Chitosan (DMEC) and Diethyl Methyl Chitosan (DEMC)

DMEC is an N-alkyl chitosan derivative prepared by the Schiff condensation reaction between amino and carbonyl groups of chitosan, followed by water removal. In particular, Bayat et al. [102], synthesized DMEC by two reaction steps, as reported by Kim, et al. [103], in order to enhance the oral bioavailability of peptides. In the first step, the authors introduced an ethylic group on the amino group of chitosan and in the second one methyl iodide was added to produce DMEC. Like all the other *N*-alkyl chitosan derivatives, DMEC has antimicrobial, anticancer and antioxidant activity and could be applied in tissue engineering [104].

The synthesis of DEMC, another *N*-alkylchitosan derivative, is similar to that of DMEC [103], but in the second step, ethyl iodide instead of methyl iodide was added. Avadi et al. [105], synthesized a DEMC with a quaternarization degree of 79%, responsible for the complete solubility of the polymer in water at room temperature. They found that DEMC has more antibacterial activity against *E. Coli* than chitosan, thanks to its high charge density that interacts with bacteria more than chitosan. The antimicrobial activity was found to be pH-dependent and an increase in the concentration of acid acetic to the medium led to a decrease in minimum inhibitory concentration (MIC) and minimum bactericidal concentration (MBC).

Like TMC, DEMC was able to enhance the absorption of hydrophilic drugs through the tight junctions both ex-vivo and in vivo [106].

Sadeghi et al. [52], compared free-soluble forms and nanoparticulate systems based on TMC, DMEC, DEMC and triethyl chitosan (TEC) for their ability to enhance insulin intestinal absorption. The absorption promoting effect was due to the ability of the polymers to open the tight junctions between cells and depended on polymer positive charge density. For this reason, the nanoparticles based on these polymers were found unable to promote absorption. On the basis of these results it could be erroneously concluded that the encapsulation of proteins in NPs is useless for their absorption. However, it should be borne in mind that the proteins administered orally in addition to having a low permeability have a poor stability which could instead be increased precisely by their encapsulation in nanosystems.

DMEC can be modified to enhance its potential in oral delivery. In fact, nanoparticles based on thiolated DMEC (DMEC-Cys) were successfully prepared to obtain buccal films for the delivery of insulin [53]. The paper reports ex-vivo studies demonstrating that DMEC-Cys nanoparticles enhanced insulin permeation (up to 97.18%) through rabbit buccal mucosa much more than unmodified chitosan and DMEC.

### 2.5. N,O-[N,N-Diethylaminomethyl(Diethyldimethylene Ammonium)nmethyl] Chitosan (QA-Ch) Derivatives

The QA-Ch derivatives were prepared by our group by an aminoalkylation reaction between chitosan and 2-diethylaminoethyl chloride. This reaction resulted in the formation of derivatives having small pendant chains containing a number, n, of adjacent quaternary ammonium groups [107]. These derivatives were studied for their ability to enhance the permeation of hydrophilic and lipophilic drugs through different membranes, such as buccal, intestinal and corneal. For this purpose, the polymer structural parameters, i.e., degree of substitution, DS, and number of quaternary ammonium groups in pendant chains, n, were modulated [108,109]. It was found that all the QA-Ch derivatives were able to promote the absorption of drugs through both the paracellular and transcellular pathways of mono- and multilayered epithelia and that the most effective had a higher positive charge density.

It is known that thiolated chitosan derivatives have a high mucoadhesivity thanks to their ability to make an exchange reaction with disulphide groups of mucus or through an oxidation reaction with cysteine residues of mucus glycoproteins both resulting in the formation of disulphide bonds between derivatives and mucus [110,111,112,113]. For this reason, since non-substituted amino groups remained on the chitosan backbone, a multifunctional derivative of chitosan, containing both quaternary ammonium and thiol groups (QA-Ch-SH) was obtained [114]. QA-Ch-SH derivatives were synthesised by covalent attachment of thiol groups on free primary amino groups of QA-Ch, via formation of 3-mercaptopropionamide moieties. These derivatives enhance the permeability of hydrophilic drugs through intestinal epithelium, and of lipophilic drugs, such as dexamethasone, through corneal epithelium, which enhanced the intraocular bioavailability of both these drugs [115]. The interaction between QA-Ch-SH derivatives and the intestinal and corneal epithelia is due to a synergism between the quaternary ammonium and the thiol groups of QA-Ch-SH.

In the light of the above information, it was thought intriguing to compare the value of the drug mean residence time in the rabbit pre-corneal area, as determined after instillation of a drug solution containing the positively charged mucoadhesive polymer QA-Ch, or QA-Ch-SH, with that for drug loaded nanoparticles prepared from the same polymer [54]. This comparison would clarify the actual advantages of formulating nano-structured aggregates of specific chitosan derivatives, rather than simple solutions of these non-aggregated polymers, to enhance the contact time of drugs administered by eye-drops, and hence their ocular bioavailability. The corticosteroid dexamethasone phosphate (DP), and the peptide met-encephalin acetate (ME) were chosen as model drugs [54]. The data obtained showed that the nanoparticles are considerably more effective than the parent mucoadhesive polymer when they concurrently adhere to the ocular surface and strongly interact with DP molecules in solution. In such cases, it may be worth developing the often complicated preparation of a stable nanoparticle dispersion. On the contrary, nanoparticles made from the mucoadhesive thiolated QA-Ch-SH, which interacted weakly with the non-entrapped DP, were approximately as effective as the non-aggregated parent polymer. In these cases, the preparation of the medicated polymer solution is a simpler, hence, more convenient way to prolong the drug corneal contact time. Quite different is the situation with the peptide ME, the poor mean residence time value of which is mainly due to enzymatic hydrolysis. Only ME entrapment in the supramolecular systems was able to shield the peptide from aminopeptidase activity to a significant extent, whereas the non-aggregated parent polymers were ineffective [54]. These results were confirmed by an NMR investigation, which showed that the DP entrapped in nanoparticles was involved in strong interactions inside them [116].

The presence of thiol groups on the QA-Ch chain seems to improve the wound healing properties of polymer, thus accelerating the healing process. Felice et al. [23], explored the efficiency of QA-Ch and QA-Ch-SH conjugates with high and low molecular weight (MW) in the regeneration of wounds and demonstrated that high MW QA-Ch-SH promoted fibroblast cell migration and accelerated wound healing.

Since thiomers undergo oxidative degradation in aqueous environment at pH higher than 5, it is important to protect thiol groups, in order to increase polymer interaction with mucosal epithelia. For this reason, protected thiolated quaternary ammonium chitosan derivatives (QA-Ch-S-pro) were synthesized by forming disulphide bonds between the thiol groups of the ligand 6-mercaptonicotinamide and the free thiol groups of the polymer QA-Ch-SH according to a procedure previously reported [55,117]. These derivatives demonstrated stronger mucoadhesivity properties than the corresponding thiolated non protected parent polymers [118].

Regarding ocular delivery, QA-Ch and QA-Ch-S-pro were used to prepare a thermosensitive ophthalmic hydrogel (TSOH) for the transcorneal administration of 5-fluorouracil [66]. The introduction of 5-fluorouracil-medicated NPs based on Ch in TSOH increased the transcorneal penetration of the drug when administered in rabbit eyes, leading to a time-constant 5-fluorouracil concentration in the aqueous for 7h from instillation. Subsequently, TSOH containing Ch-nanoparticles was compared with TSOH containing QA-Ch nanoparticles in order to study the impact of the surface characteristics of nanoparticles on 5-fluorouracil bioavailability [67]. The instillation, in rabbit eyes, of TSOH containing NPs based on QA-Ch or sulfobutyl chitosan (SB-Ch) led in both cases to a plateau of the concentration in the aqueous humour for 10 h. Negative charges on the surface of SB-Ch-based NPs slowed down the release of 5-FU from TSOH, while the positive charges of the QA-Ch-based NPs increased NP contact with the negatively charged ocular surface. Both resulted in a higher ocular bioavailability [67].

Recently, QA-Ch and QA-Ch-S-pro-based NPs were prepared to study the effect of mucoadhesion on oral bioavailability of a protein model drug (FD4) [55]. In vivo data obtained with rats for the FD4 plasma concentration vs. time pattern showed that the bioavailability of FD4 was higher when this was administered via QA-Ch-S-pro-based NPs than with QA-Ch-based NPs. Moreover, the peak time moved from 1 to 2 h. The bioavailability increase was ascribed to an increased NPs residence time at the absorption site, associated with an increase in NPs adhesion to the mucus lining of the intestinal epithelium [55].

It is known that the poor solubility of drugs in physiological fluids may be responsible for their poor absorption and, therefore, for the insufficient bioavailability of the drug. For this reason, the solubilization ability of metyl-β-cyclodextrin (MCD) and the mucoadhesive properties of QA-Ch were merged into a QA-Ch-MCD derivative. The macromolecular product (QA-Ch-MCD) and its relevant nanoparticulate carrier (NPs) were thoroughly characterized and compared in terms of their ability to promote the absorption of the poorly soluble model drug dexamethasone (DEX) [65]. In vitro and ex-vivo studies revealed a stronger mucoadhesivity of the macromolecular complex, resulting in a more difficult transport through mucus, with respect to NPs. Drug permeation through excised rat intestine was faster when the macromolecular complex was used as the carrier. Meanwhile, the permeation rates of the fluorescein isothiocyanate (FITC) labelled carriers were comparable. Then, the use of NPs did not seem to provide any determinant advantage over using the simpler macromolecular complex [56]. The role of the QA-Ch-MCD conjugation concerning the conjugate ability to bind the dalargine peptide (DAL) in comparison with that of the physical mixture of QA-Ch and MCD was also investigated. The data showed a greater ability of QA-Ch-MCD to protect DAL from degradation by α-chymotrypsin compared to the physical mixture of the precursors. This ability can be attributed to a synergistic cooperation of cyclodextrin and polymer, which occurs only when the former is covalently linked to the latter [57].

Many agri-food extracts are important sources of polyphenols, that is, molecules of high interest thanks to an ample variety of biological activities [119]. However, a low bioavailability is the major problem of using antioxidants from agri-food extracts in the therapy. The poor intestinal absorption, along with oxidation in GI and marked metabolism in liver make it unlikely that high concentrations of these antioxidants are found in the organism for long after ingestion, and reach the blood, that is the action site [56]. For this reason, QA-Ch and QA-Ch-SH were used to prepare nanoparticles that were tested for their ability to enhance grape seed extract oral absorption [59,60]. 

**Figure 2:**
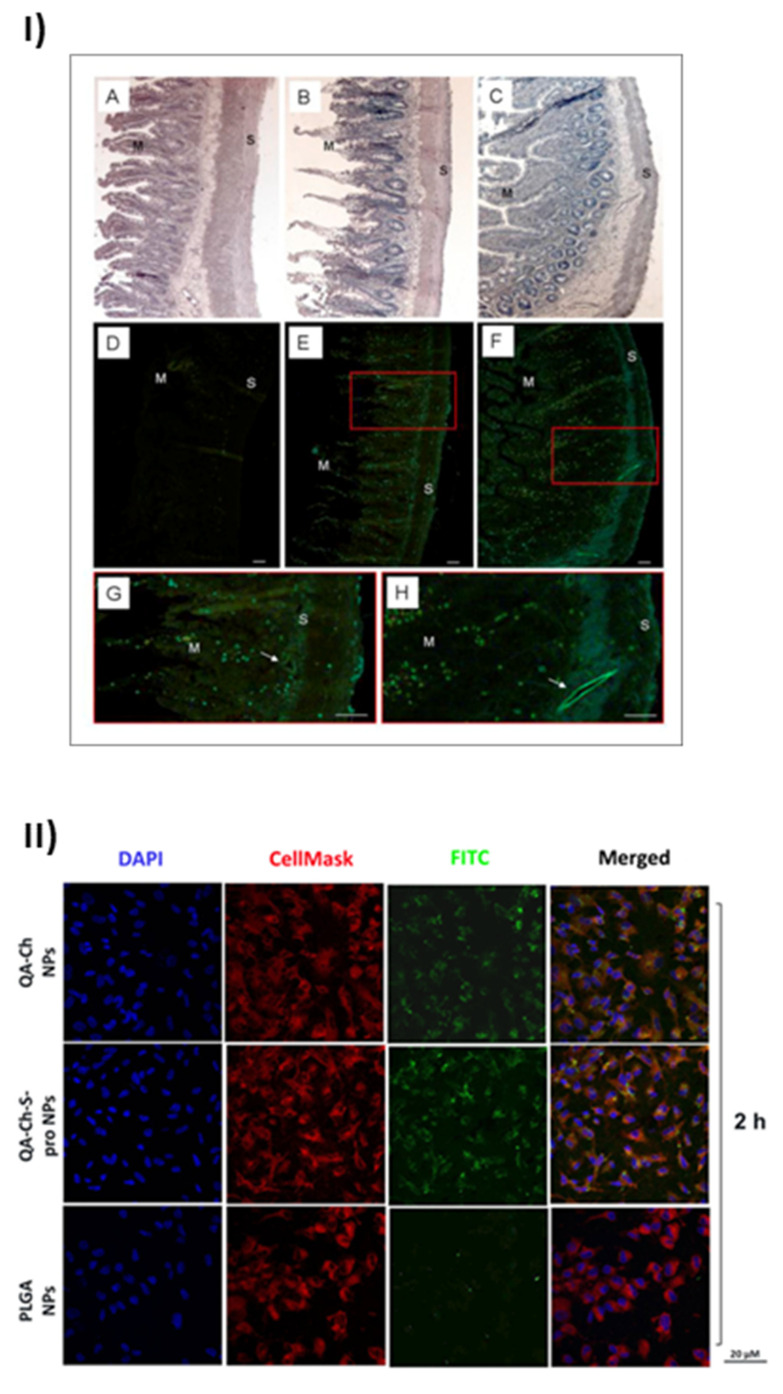
Results of the positive effects of QA-Ch and its derivatives, taken from [61] and [63]. (**I**) Microphotographs of slices of rat intestinal wall observed under a light (**A**–**C**) or fluorescence (**D**–**H**) microscope following 1h incubation in an Ussing chamber with sodium fluorescein (NaFlu) solution (**A**,**D**); FITC-QA-Ch-based NP (**B**,**E**,**G**) or FITC-QA-Ch-SH-based NP (**C**,**F**,**H**). Haematoxylin-eosin stained sections (**A**,**B**) show intact intestinal epithelium. In the control sample **(D)** no florescence is visible, while some fluorescent spots are present in FITC-NP treated samples (**E**–**H**). The fluorescent spots are visible all across the gut section, from mucosal (M) to serosal (S) layer and at the level of vessels (arrows) (**G**,**H**). **G** and **H** are a larger magnification of boxes in **E** and **F**, respectively. Scale bar 50 μm. (**II**) Confocal fluorescence microscopy analysis of human umbilical vein endothelial cells (HUVEC) incubated with FITC-labeled QA-Ch, QA-Ch-S-pro or PLGA NP (green color) for 2 h. CellMask and DAPI for staining the cellular membrane and nucleus are shown in red and blue colors, respectively. Scale bars are 20 µm. Control is represented by untreated cells.

The uptake of nanoparticles by endothelial progenitor cells (EPC) upon incubation with grape seed extract loaded in FITC-labelled NPs based on QA-Ch or QA-Ch-SH was studied. Both NP types were partially internalized by cells, QA-Ch-based NPs being seemingly taken up to a higher extent than QA-Ch-SH-based NPs. This difference can safely be correlated with the stronger positive surface charge of the former, resulting from zeta-potential measurements. Moreover, it was found, as shown in Figure 2I, that following incubation of NPs dispersions with the mucosa of excised rat intestine, NPs migrated from donor to acceptor compartment and penetrated across the intestinal wall in an integral state [61]. Recently an analogous study with autochthonous cherry extracts from the Region Tuscany was carried out. In this case the extracts were encapsulated in NPs based on two different polymer types, namely QA-Ch and QA-Ch-S-pro. The data obtained showed that the cherry extracts encapsulated in nanoparticles are much more stable than the non-encapsulated ones and are not degraded in the gastric environment [62]. NPs from both types of chitosan derivatives promoted the absorption of cherry extracts with no significant difference between the two nanoparticle types. The same nanoparticles were compared to those prepared from poly(lactic-co-glycolic acid) (PLGA) [63]. All nanoparticle types were able to promote the permeability of encapsulated extract and showed good anti-inflammatory activity. However, as shown in Figure 2II, NPs prepared from Ch derivatives were more capable of being internalized by endothelial cells than PLGA nanoparticles due to the positive charges present on the surface of the former. Interestingly NPs prepared from QA-Ch-S-pro were more effective in improving the ability of cherry extract to protect endothelial cells from oxidative stress, thanks to the intrinsic antioxidant properties of the protected thiol group present on the polymer [64].

## 3. In Vitro Studies to Characterize Drug Delivery Systems Based on Quaternary Ammonium Chitosan Derivatives

In vitro studies are commonly used to establish the efficacy of a drug delivery system and predict in vivo behaviour. Jug et al. [120], reported that the future development of novel mucosal drug delivery systems can be facilitated by using the vitro-in vivo correlation mathematical model (IVIVC), which can predict the in vivo performance of a drug starting from the in vitro model used for drug release. Different in vitro models have been developed to study the effectiveness of a specific delivery system and include cellular studies, drug release studies, and evaluation of positive biological effects. The choice of one in vitro model rather than another mainly depends on the absorption route of the drug contained in the system, hence on the absorption site. In the next sections the in vitro studies of the drug delivery systems based on quaternary ammonium chitosan derivatives that have been used will be reviewed.

### 3.1. Release Studies

Although in vitro drug release tests were initially developed for solid oral dosage forms and reported by all the Pharmacopoeias, many changes have been proposed in order to study drug release from innovative delivery systems [120]. To evaluate the in vitro release of insulin from HACC microparticles coated with Eudragit L100-55, Sonia et al. [121], suspended the formulations in buffer solutions (pH 1.2 or 7.4) for 6 h, after which the insulin content was quantified by the Lowry protein assay. Due to the hydrophilic nature of HACC, at pH 7.4 the microparticles showed a burst release of insulin followed by a slow and sustained release.

On the other hand, the amount of insulin released at gastric pH was rather low thanks to the gastro retentive coating. Similarly, Xu et al. [122], studied the release profile of the model protein drug, bovine serum albumin (BSA), from HACC NPs. BSA-loaded HACC NPs were placed in test tubes with 6 mL of 0.9% (*w*/*v*) sodium chloride saline and incubated at 37 °C under stirring. After collection of samples at different times, the amount of BSA released from the nanoparticles was evaluated by the Coomassie Blue protein assay. The in vitro release profile showed four different phases, e.g., (1) an initial burst desorption of BSA from surface, (2) a 12 h BSA re-adsorption onto nanoparticle surface, (3) a plateau phase for the subsequent 3 days, resulting from diffusion of the drug dispersed in the polymer matrix, and finally (4) a constant sustained release of the drug, resulting from both protein diffusion through polymer and polymer erosion. This profile described a slow and continuous release of BSA and confirmed the potential of HACC NPs to control protein release.

In the perspective of a potential mucosal vaccine, Zao et al. [123], designed HACC and N,O-carboxymethyl chitosan (CMC) nanoparticles loaded with Newcastle Disease Virus Fusion gene plasmid DNA with C3d6 molecular adjuvant. In vitro release of plasmid DNA was performed and the results exhibited an initial burst effect followed by a prolonged and sustained release indicating the HACC-CMC nanoparticulate system as a carrier for the delivery of plasmid DNA via the nasal route [123].

A noteworthy study reported the release of diclofenac sodium (DC) from TMC nanoparticles for ocular delivery. The pH effect of the NPs reconstitution buffer was the focus of this in vitro experiment. Three different pH (5.5, 6.5 and 7.4) phosphate buffer solutions were used separately to reconstitute the NP dispersions from the lyophilized products. The receptor compartment, containing a phosphate buffer solution pH 7.4, was separated from the donor compartment through a cellulose dialysis membrane. Eight hours after the start of the experiment the NP dispersion reconstituted with 5.5 phosphate buffer showed a constant release pattern avoiding the rapid DC dissolution that instead occurred with 6.5 or 7.4 phosphate buffer reconstitution. Because of the DC pKa close to pH 5.5, the 5.5 phosphate buffer could be considered preferable as the reconstitution solution for TMC nanoparticles [124].

Meng et al. [34], designed targeted PLGA NPs coated with lactoferrin (Lf)-conjugated TMC medicated with huperzine A (HupA). These NPs were studied as a nose-to-brain delivery system for Alzheimer disease therapy. The in vitro study of HupA release was carried out using an NPs dispersion in PBS pH 7.4 placed in a cellulose membrane dialysis bag as the donor compartment, immersed in PBS pH 7.4 at 37 °C for 96 h. A prolonged and sustained release of HupA was observed, suggesting that these NPs can represent a model for further investigation in nose-to-brain delivery.

However, it is important to underline that in the case of NPs, dialysis experiments might not be descriptive of drug release, because the process could be mainly controlled by drug permeation across the dialysis membrane [125]. This view was supported by the results obtained in a subsequent work by our research group where the release of dexamethasone sodium phosphate or metenkephalin from two NPs types, based on QA-Ch or QA-Ch-SH was investigated. A dialysis method was used where equal samples of the NP dispersion under study were dialyzed for different time intervals, at the end of which each sample was ultra-centrifuged and the relative surnatant analyzed for the drug. This procedure allowed the building of the drug released from NPs vs. time graph [54]. All nanoparticle types showed an initial burst release followed by no further drug release from the nanoparticle matrix over 24 h. These results were in agreement with those found by Uccello-Barretta et al. [116]. Similar results were obtained using nanoparticles based on QA-Ch-S-pro, loaded with FD4 [104] or based on QA-Ch-CD [56].

### 3.2. Evaluation of Antibacterial, Antifungal, Antimicrobial and Antioxidant Activity

Quaternary ammonium chitosan derivatives have antioxidant effects due to the presence of the fixed positive charge. The method frequently used to assess the antioxidant activity of quaternary ammonium derivatives is the 2,2-diphenyl-1-picrylhydrazyl hydrate (DPPH) radical scavenging assay that is based on the measurement of the scavenging capacity of antioxidants [126]. DPPH method has undergone various modifications, but the basic approach remains the same: the compound under analysis is mixed with a DPPH solution and the absorbance, measured colorimetrically, is directly related to the antioxidant activity. In vitro bacteria or fungi cultures are prepared in Petri dishes or 96-well plates to study the antibacterial and antifungal activity of polymers, which are generally reported as minimum inhibitory concentration (MIC), minimum bactericidal concentration (MBC) and minimum fungicidal concentration (MFC), respectively.

Different HACC derivatives were compared for their antibacterial and antifungal activity through a microdilution broth method and evaluation of MIC [72]. Since either bacterial or fungal cell membranes are anionic due to the presence of phospholipids, cationic polymers can interact with the negative charges and cause membrane damage, leading to cell death. In vitro studies confirmed this mechanism of action for HACC derivatives against bacteria or fungi.

Similarly, to evaluate the antimicrobial and antibacterial activities of DEMC, Avadi et al. [105], analyzed the MIC by a turbidimetric method, consisting in mixing different test tubes containing DEMC and *E. coli* suspensions and studying them for visible signs of growth or turbidity. Subsequently, MBC was evaluated by inoculating the live organisms that had shown no growth in the MIC test on Eosin-Methylene Blue and looking again for signs of growth. The final results showed that DEMC has a higher inhibitory effect against *E. Coli* and a higher antibacterial activity than chitosan, thanks to the high charge density through which it interacts with bacteria.

To investigate the antibacterial activity of TMC NP/chitosan composite sponge against *E. Coli* and *S. aureus*, Xia et al. [127], employed the transwell methods. A bacterial suspension was added to 12-well plates basolateral chamber, whereas the materials under test were placed in the apical chamber of a transwell plate for 24h. Serial dilution of bacteria culture were plated on Lysogeny broth (LB) agar plate and counted. The results showed a more intense antibacterial activity of TMC NP/chitosan with respect to chitosan alone. This was ascribed to the presence of several quaternary ammonium groups on the TMC NPs surface, which enhanced the electrostatic interaction with the anionic charges of bacteria components.

TMC/poly(vinyl alcohol) (PVA) hydrogels cross-linked with glutaraldehyde were studied in vitro for biodegradation, uptake and swelling ability and antimicrobial activity. The hydrogel samples were immersed in a simulated body fluid solution at pH 7.4, in order to study biodegradation and swelling ability at different times, up to 192 h. The antimicrobial test for Gram-positive and Gram-negative bacteria and fungi was carried out using the agar well diffusion method by measuring the inhibition zones against the test organisms. Moreover, MIC was determined by micro-dilution method in 96-well plates. The results showed that PVA increased hydrogel swelling ability and that the cross-linking with the highest percentage of glutaraldehyde reduced hydrogel biodegradation and enhanced hydrogel antimicrobial activity [92].

Li et al. [128], compared different quaternary ammonium derivatives containing pyridine or amino-pyridine and demonstrated that the position of the amino group on pyridine can influence their antioxidant properties. In fact, amino group on the C-3 position of the pyridine ring has an important role on the scavenging activity against hydroxyl radicals and DPPH-radicals. This group acts as an electron donor able to quench and stabilize reactive free radicals, thus showing high influence on the antioxidant activity.

Antimicrobial and bactericidal activities of *N*-quaternary ammonium-*O*-sulfobetaine-chitosan cotton fabrics against Gram-negative bacteria *E. coli*, Gram-positive bacteria *S. aureus* and the fungus *C. albicans* were evaluated by Zhang et al. [129], using the viable cell count quantitative method. The results from all studies show that cotton fabrics have a strong antimicrobial activity against *S. aureus* than *E. coli*. In fact, the synergistic effect of quaternary ammonium chitosan and sulfobetaine can generate reactive oxygen species which are more destructive for *S. aureus*.

Wei et al. [21], evaluated the antioxidant activity of 6-*O*-imidazole-based quaternary ammonium chitosan derivatives through three in vitro radical scavenging assays, including DPPH, hydroxyl, and superoxide radicals. The authors also performed antifungal assays to determine the minimum inhibitory concentration and the mycelium growth inhibition. In all cases, the derivatives showed higher antioxidant activity than chitosan, due to the higher density of positive charge and the strong electron donor ability of the substituent which contribute to their bioactivity. All these studies show how important the positive fixed charge is for the antimicrobial activity of the polymers, however it must also be considered that at the neutral pH of the culture broths the chitosan is insoluble and that its lower activity can also depend on this aspect.

### 3.3. Evaluation of the Nanosystems Ability to Diffuse through Mucus

Different study methods have been developed to evaluate the nanoparticles transport through the mucus [130,131,132,133,134]. The most used method one is the multiple particle tracking [135], based on analysis of the diffusion of fluorescent NP into the mucus as observed by a microscope equipped with a camera. However, this method does not take into account the water movement through the mucus which could influence NPs diffusion. Inside the intestine there are two different water movements, the first one is longitudinal, responsible for transporting NPs away from the absorbent epithelium, the second one is transverse, from the gastrointestinal lumen to the absorbent epithelium and vice versa. Indeed, the fundamental task of the intestine is the absorption of nutrients and salts which implies water absorption to balance blood osmolarity. The transverse water movement in intestinal mucus is advective, that is, the water and dissolved substances are transported by bulk motion.

Recently we developed a simple method for studying the ability of nanosystems to penetrate through mucus [136]. Using this method, we succeeded in simulating the advective water movement from intestinal lumen to epithelium across the mucus lining. This method has appeared more predictive than, for example, the multiple particle tracking method. Indeed, with the latter the transport of nanoparticles in mucus is observed in the absence of the water movement which, in fact, can be decisive in pushing particles through the mucus to reach the epithelium, that is the absorption site. Two types of nanoparticles loaded with fluorescein isothiocyanate–dextran 4 KDa (FD4) were tested with this method: QA-Ch-based NPs QA-Ch-S-pro-based NPs. QA-Ch-S-pro NPs resulted more mucoadhesive than those prepared from QA-Ch, hence less able to diffuse in the mucus.

FD4 plasma concentration-time profiles and the corresponding pharmacokinetics parameters founds in vivo in rats showed that NP QA-Ch-S-pro-based NPs had a significantly higher bioavailability than QA-Ch-based NPs [55]. The results of this work indicate that drugs trapped in the more mucoadhesive NP type have a higher oral bioavailability than those trapped in less mucoadhesive ones. Indeed, mucoadhesivity tends to keep the formulation at the absorption site, while water movement facilitates NP transport across the mucus layer from lumen to epithelium where NP can be internalized by cells.

### 3.4. Cell Studies

The biocompatibility assessment of biomedical polymers is essential to ensure the safety of systems intended for use. The biological evaluation of medical devices, in terms of the procedures to identify and quantify the biological risks associated with the use of biomedical materials, is governed by the International Organization for Standardization (ISO) 10993 in Europe (ISO 10993) and by the Food and Drug Administration (FDA) blue book memorandum G95-1 in the United States (#95-1, US FDA). The biocompatibility of polymers remains one of the key aspects that must be investigated because it is the major factor limiting their use in the biomedical field. In vitro cell cultures represent a powerful tool for the preliminary screening of biomaterial cytotoxicity. Cell-based assays can provide essential information on the potential effects of chemicals on specific cell properties and provide sound basis for further molecular studies [137]. Although chitosan is considered non-toxic and biologically biocompatible, its modifications could make it more or less toxic, hence the biological effects of chitosan derivatives should be investigated.

#### 3.4.1. Oral/Intestinal

Usually, the Caco-2 cell monolayer is the most used model for studying the ability of mucosal delivery systems to improve intestinal absorption [138]. However, the research is increasingly moving toward cell models that are more capable of mimicking the epithelial/endothelial barriers characterizing a specific mucosal surface. Indeed, innovative cell models including co-culture and triple co-cultures, e.g., Caco-2/HT29, Caco-2/Raji-B and Caco-2/HT29/Raji-B), have been recently developed for studying intestinal permeation and cell uptake of nanoparticles with different physical-chemical characteristics [139].

The triple co-culture model Caco-2/HT29-MTX/Raji B was used by Beconcini et al. [63], to study the oral permeability of QA-Ch and QA-Ch-S-pro nanoparticles loaded with cherry extracts. The model was prepared by a previously described and validated method [140]. Briefly, Caco-2 and HT29-MTX cells were seeded together into a transwell insert, subsequently Raji B were added to the basolateral compartment, and the transepithelial electrical resistance (TEER) was monitored starting from the second week of co-culture. It was observed that only the NPs based on QA-Ch-S-pro significantly promoted the permeation of the encapsulated cherry extracts, thanks to a stronger adhesion to the mucus layer with respect to the less mucoadhesive QA-Ch-based NPs.

The in vitro cytotoxicity of TMC- and CMC/TMC-liposomes was investigated through CCK8 assay using mouse fibroblast cells (L929) and human colorectal adenocarcinoma cells (Caco-2) [42]. After 24 h of liposome incubation with both cell types, a concentration-dependent cell viability was observed. TMC liposomes showed a higher cytotoxicity than CMC/TMC-liposomes and control, due to an electrostatic interaction between the positively charged TMC and the negatively charged cell membranes, which can cause cell rupturing. Then the CMC/TMC liposomes appeared to be the most cytocompatible.

#### 3.4.2. Pulmonary

The new bronchial epithelial cell line VA10 was used for the evaluation of different quaternary ammonium chitosan derivatives, including TMC, as drug permeation enhancers and for possible pulmonary applications. In particular, the authors studied the ability of these derivatives to promote the paracellular transport of the macromolecular marker FD4 [88]. Since all derivatives, TMC in particular, caused a dose dependent decrease in TEER and an increase in paracellular permeability of FD4, they could be used to increase the paracellular permeation of hydrophilic macromolecules such as peptide and protein-based drugs.

#### 3.4.3. Skin/Transdermal

Recently, Xia et al. [127], tested the cytotoxicity of TMC NPs/CS composite sponge by 3-(4,5- dimethylthiazol-2-yl)-2,5- diphenyltetrazolium bromide (MTT) assay using L929 cells for 48 or 72 h. The asymmetrically wettable TMC NPs/CS composite sponge exhibited no toxicity, thus suggesting its use as an excellent material for wound dressing. 

In a research carried out by Felice, et al. [23], primary fibroblasts cell viability was tested by WST-1 assay in the presence of quaternary ammonium–chitosan conjugates and their thiolated derivatives with high or low molecular weight. All chitosan derivatives showed a complete biocompatibility at a concentration of 10 μg/mL for 24 h. Wei et al. [21], assessed HaCat cell keratinocytes viability by testing 100 μg/mL 6-*O*-imidazole-based quaternary ammonium chitosan derivatives by the MTT assay. The test exhibited values of cell viability up to 80% suggesting a good biocompatibility of the developed system.

HACC modified with nisin was studied for cytotoxicity on NIH-3T3 fibroblasts from mouse by using MTT assay [141]. After 24 h and 48 h cell incubation, HACC showed a low cytotoxicity but a reduced cell viability with respect to unmodified HACC, because of the introduction of nisin, which may damage the structure of glycoproteins on the NIH-3T3 cell membrane. However, the results demonstrated that HACC modified with nisin could be a promising candidate for wound dressing application if used in a certain concentration range.

#### 3.4.4. Ocular

To assess the eye irritation of TMC NPs loaded with diclofenac sodium, confluent rabbit corneal cell line (SIRC) were incubated with a solution prepared from lyophilized TMC NPs (0.5–5%) following the short time exposure (STE) [124] validated by Takahashi et al. [142]. The treated cells were analysed by the MTT assay and the resulting score revealed that the TMC NPs were safe.

#### 3.4.5. Nasal

Karavasili et al. [143], developed PLGA/1,2-dipalmitoyl-sn-glycero-3-phosphocholine (DPPC)/TMC polymer-lipid microparticles loaded with ropinirole hydrochloride for the treatment of Parkinson disease via intranasal delivery. Concentrations of 50–1000 μg/mL of the sample were tested on human lung epithelial cells Calu-3 using MTT assay revealing the safety of the microparticles.

Meng et al. [34], studied in vitro cell viability of 16HBE cells taken as a model of nasal mucosa for nose-to-brain delivery system for Alzheimer disease therapy. MTT was carried out for 24 h and no toxicity was observed of PLGA nanoparticles conjugated with TMC up to a concentration of 10 mg/mL of TMC.

### 3.5. Ex-Vivo Studies

Ex vivo tests are important as well as in vitro tests for the development of a controlled release bioadhesive system because they contribute to studying permeation, compatibility, mechanical and physical stability, superficial interaction between formulation and mucous membrane and strength of the bioadhesive bond [144]. Therefore, different types of ex-vivo models have been used to evaluate the mucoadhesive properties of oral, buccal, periodontal, nasal, gastrointestinal, vaginal or rectal delivery systems [145]. However, the most reliable ex-vivo methods combine the study of mucoadhesive and permeation properties to gain useful information about the behaviour of the drug delivery systems.

In order to evaluate the potential of NPs to facilitate insulin transport, Yin et al. [94], monitored the transport of insulin either free or contained in TMC or TMC-Cys nanoparticles through rat ileum. The formulations were incubated with the ileal loop. Subsequently the ileal loop was washed with saline and the remaining concentration of insulin was quantified. In order to evaluate the ability of nanoparticles to facilitate insulin transport, the formulations were syringed into the rat ileal sac and the insulin concentration in the incubation buffer (Kreb’s-Ringer buffer) was quantified at different time intervals. It was found that TMC-Cys nanoparticles, thanks to the presence of mucoadhesive thiol groups, enhanced insulin permeation more than TMC nanoparticles. This result was confirmed by an in vivo mucoadhesion study.

Another reported method based on the use of the excised rat intestine was used to study the permeation characteristics of a drug delivery system. Briefly, excised rat intestine, accurately washed, was mounted in an Ussing type chamber and the transport of the drug from apical to basolateral side was measured at different time intervals [114]. To study the mucoadhesive properties of a drug delivery systems the excised rat intestine was used. This was cut into segments and incubated 3 h with the formulations to be tested. Using this method, the authors demonstrated that nanoparticles based on QA-Ch-SH were more mucoadhesive than nanoparticles based on QA-Ch due to the presence of thiol groups on the former [59].

Both these methods were used to demonstrate the ability of mucoadhesive QA-Ch and QA-Ch-S-pro derivatives to improve the intestinal permeation of cherry extracts (CE). The results showed that, although QA-Ch-S-pro nanoparticles were more mucoadhesive than QA-Ch nanoparticles, both nanoparticle types were able to promote the intestinal absorption of CE and protect CE polyphenols from degradation in the stomach, thus increasing their oral bioavailability [62,63].

## 4. Concluding Remarks

In this review, the relevance of the fixed positive charge, present on the quaternary ammonium chitosan derivatives, to the preparation of drug release systems was highlighted.

Many innovative systems have been prepared with these polymers, such as thermosensitive hydrogels, polymeric nanoparticles, liposomes, nanocomplexes for the administration of drugs, nutraceutical products or genes through different routes of administration or for local application.

These systems have shown excellent characteristics thanks to the positive fixed charge on the polymer backbone, which resulted in improved mucoadhesiveness, antibacterial and antimicrobial properties, enhancement of drug absorption through both the transcellular and paracellular pathways, and promotion of wound healing compared to unmodified chitosan-based systems. The nanosystems prepared with these polymers have also shown an improved ability to be internalized by a wide variety of cells, thus promoting the absorption of the encapsulated drugs. This effect was related to the positive charge density present on the surface of nanosystems. Certainly it must be taken into account that the cytotoxicity tests have shown a greater cytotoxicity of the quaternary ammonium derivatives compared to unmodified chitosan, however these polymers can be used safely within a fairly wide range of concentrations.

## Figures and Tables

**Table 1:** Overview of drug delivery systems based on quaternary ammonium chitosan derivatives.

Quaternary Ammonium Chitosan Derivatives	Drug Delivery System	References
N-2-hydroxypropyltrimethyl ammonium chloride chitosan (HACC) 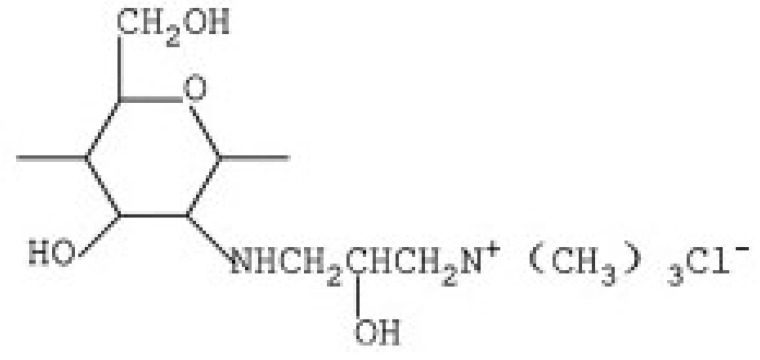	Wound dressings Hydrogels Nanoparticles	[24] [25,26] [27,28,29,30]
N-trimethyl chitosan (TMC) 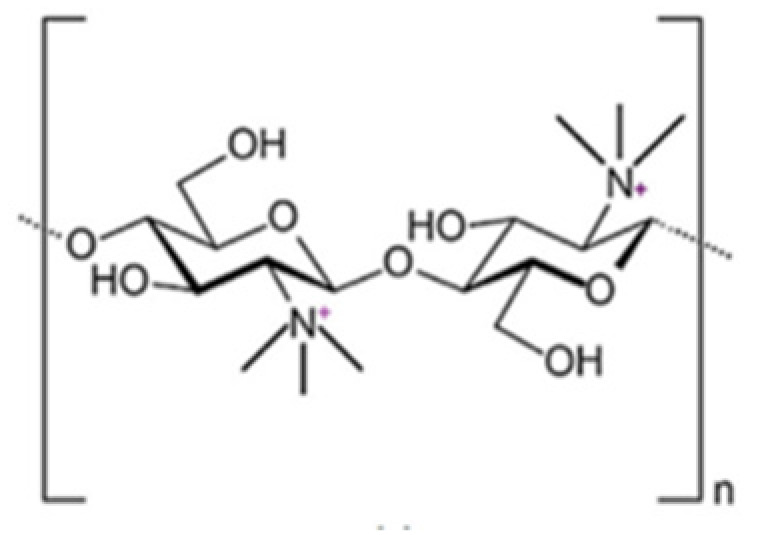	Nanoparticles Polyelectrolyte complexes Liposomes Wound dressings Hydrogels Nanocomplexes Polyplexes	[31,32,33,34,35,36] [37] [38,39,40,41,42] [43] [44] [45,46,47] [48]
Quaternary carboxymethyl chitosan (QCMC) 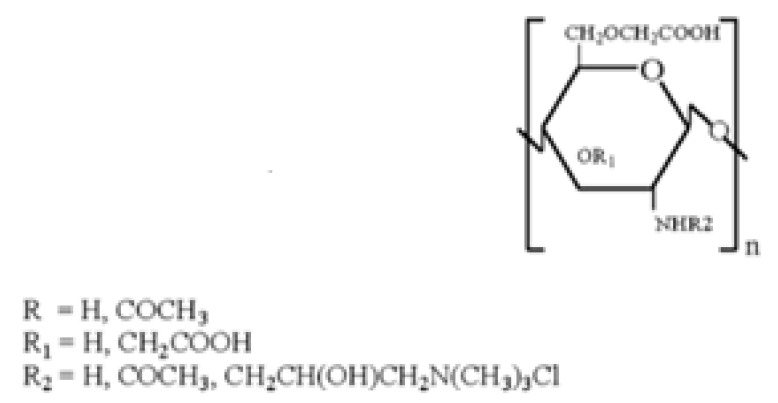	Films Nanoparticles Liposomes	[49] [50] [51]
Dimethyl ethyl chitosan (DMEC) and diethyl methyl chitosan (DEMC) 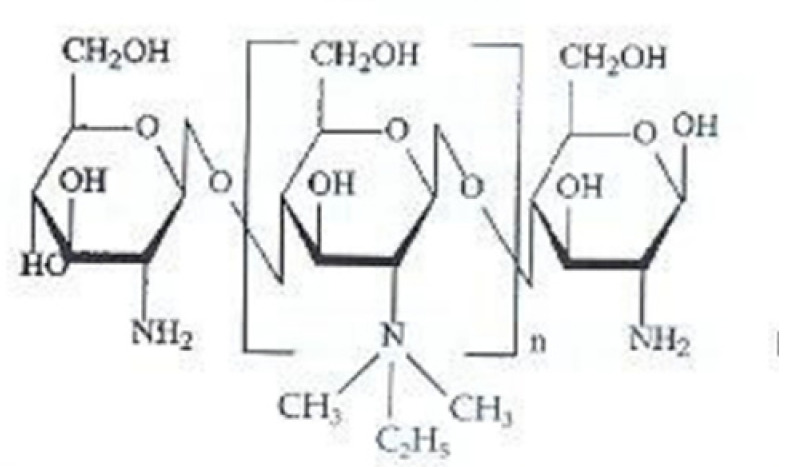 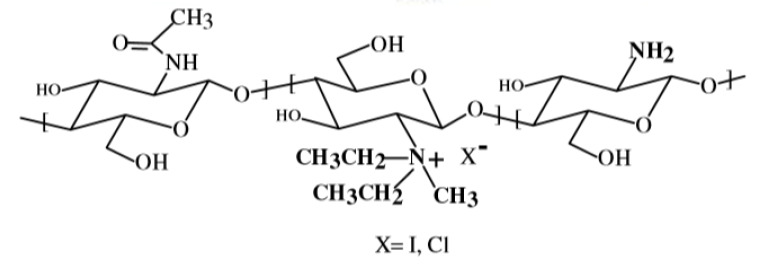	Nanoparticles	[52,53]
N,O-[N,N-diethylaminomethyl(diethyldimethylene ammonium)nmethyl] chitosan (QA-Ch) 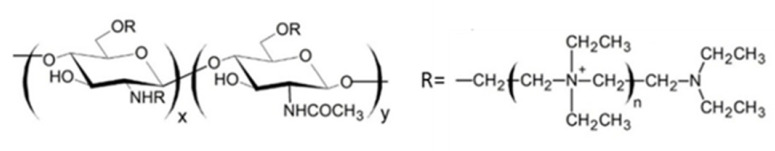 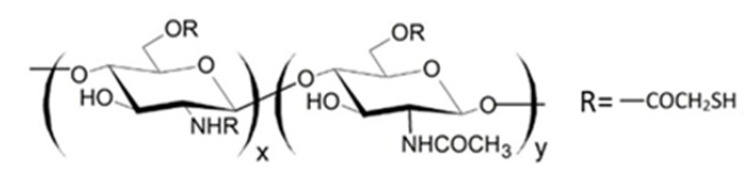 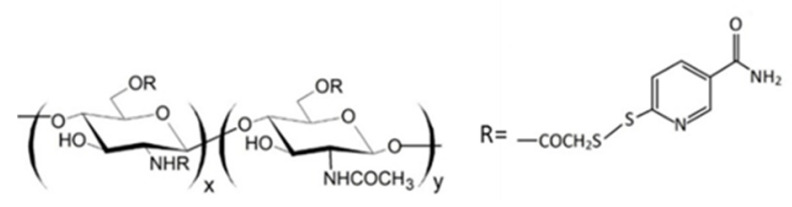	Nanoparticles Nanocomplexes Hydrogels Wound dressings	[54,55,56,57,58,59,60,61,62,63,64] [65] [66,67] [23]
